# Non-invasive vagus nerve stimulation for the treatment of cluster headache: a case series

**DOI:** 10.1186/1129-2377-1-S14-P231

**Published:** 2013-02-21

**Authors:** AD Nesbitt, JCA Marin, E Tomkins, MH Ruttledge, PJ Goadsby

**Affiliations:** 1 Royal Free London NHS Foundation Trust, UK; 2 Beaumont Hospital, Dublin, Ireland

## Introduction

Cluster headache is a highly disabling primary headache syndrome. A need exists for a novel, safe and practical approach to add to the current therapeutic armamentarium.

We assessed the usefulness of a new non-invasive, portable vagus nerve stimulation (VNS) device, the GammaCore, which has been developed for the treatment of headache.

## Methods

Patients attending headache centres in the UK and Ireland were offered non-invasive VNS treatment in an unbiased fashion. Case notes were reviewed, and patients questioned during routine follow up about their experience with the device and the impact they perceived it had.

## Results

Of patients given the device 14 of 17 (9 male; 7 chronic, all medically intractable, 7 episodic; median age 46, range 13-84) had sufficient data available during a median device use period of 13 weeks (range 2-26) to include in analysis. Thirteen felt there was an overall improvement in their condition since using the device, stating a mean estimated subjective improvement of 60% (SD 30) from baseline. One patient’s condition remained the same. Seven were able to reduce significantly or stop their previous abortive treatment, five had reduced it and two required the same amount as previously. Five were very satisfied, eight satisfied and one equivocally satisfied after using the device. All 14 would recommend the treatment to others.

Ad hoc analysis suggests that treatment can both abort and significantly improve attacks, as well as having secondary effects of reducing attack frequency when used prophylactically.

## Conclusions

This is the first evaluation of an entirely new treatment modality for cluster headache. We have developed a paradigm that appears to be well-tolerated and effective in both the acute and preventive treatment of episodic and chronic cluster headache.

These preliminary findings suggest a formal clinical trial is warranted to further establish the efficacy of this treatment.

## Conflict of interest

This work was supported by an unrestricted grant from ElectroCore Medical, LLC.

